# Supplementary material for: The Impact of Circular Exercise Diameter on Bone and Joint Health of Juvenile Animals
Source: Animals (Basel). 2022 May 27;12(11):1379. doi: 10.3390/ani12111379 (PMC9179390; doi:10.3390/ani12111379)
Supplement: Supplementary file 1 [file animals-12-01379-s001.zip › animals-1725809-supplementary.pdf]

Figure S1: Equation utilized to calculate bone mineral density (BMD) of values measured in computed tomography (CT) scans

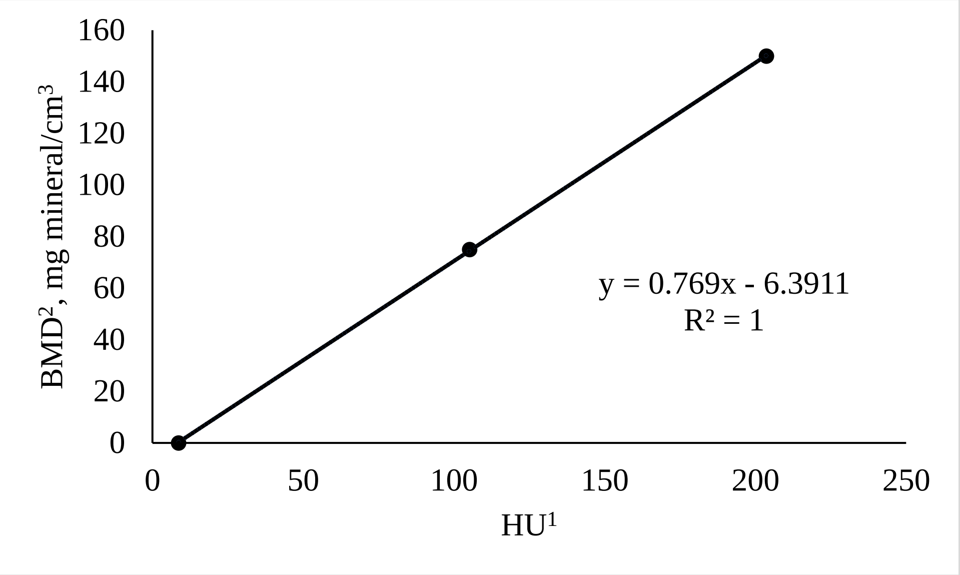

[1]Hounsfield Units (HU): Values along the x-axis are average HU values obtained from CT scans

[2]Bone Mineral Density (BMD): Values along the y-axis are known concentrations of rows in the hydroxyapatite phantom (0, 75, and 150 mg mineral/cm^3)

Table S1: Calf height, weight, and length expressed throughout the weekly measurements

Day	Height (cm)	Weight (kg)	Length (cm)
0	88[g]	78[g]	89[e]
7	90[f]	90[f]	89[e]
14	92[e]	87[f]	95[d]
21	96[d]	108[e]	95[d]
28	96[d]	119[d]	103[c]
35	98[c]	130[c]	104[c]
42	99[b]	140[b]	109[b]
48	100[a]	148[a]	113[a]
SEM	1	3	1
P – Value	$P < 0.001$	$P < 0.001$	$P < 0.001$

[a,b,c,d,e,f,g] Values lacking common superscripts within a column differ ($P < 0.001$)

Table S2: Internal (int) and external (ext) dorsopalmar and mediolateral diameters as well as moment of inertia (MOI) from cross-sectional views at the midpoint of fused metacarpal III & IV of left and right front legs

Treatment	Dorsopalmar int, mm	Dorsopalmar ext, mm	Mediolateral int, mm	Mediolateral ext, mm	MOI, mm^4
Control	12	21	17	29	11,600
Large	12	21	19	29	11,200
Small	12	22	18	30	12,900
Treadmill	12	21	17	28	11,500
SEM	0.40	0.39	0.52	0.61	820
P – Value	0.60	0.27	0.16	0.29	0.50

Table S3: Cortical and midpoint slice bone density of metacarpal III & IV (MC III & IV) of left and right front legs

Treatment	Dorsal cortex, mg mineral/cm3	Lateral cortex, mg mineral/cm3	Medial cortex, mg mineral/cm3	Palmar cortex, mg mineral/cm3	Midpoint slice, mg mineral/cm3
Control	1,240	1,220	1,220	1,060	1,030
Large	1,240	1,240	1,230	1,070	1,030
Small	1,250	1,230	1,240	1,070	1,040
Treadmill	1,250	1,230	1,240	1,060	1,030
SEM	21	12	14	17	11
P – Value	0.95	0.73	0.76	0.97	0.96

Table S4: Cortical widths at midpoint of the metacarpal III & IV (MC III & IV) of left and right front legs

Treatment	Dorsal cortex, mm	Lateral cortex, mm	Medial cortex, mm	Palmar cortex, mm
Control	5.0	5.6	5.7	4.1
Large	5.0	5.2	5.2	3.9
Small	5.4	5.4	5.7	4.0
Treadmill	5.2	5.4	5.5	4.2
SEM	0.20	0.31	0.28	0.21
P – Value	0.38	0.86	0.63	0.70

Table S5: Dorsal, lateral, medial, and palmar cortical widths of the metacarpal III & IV separated by left and right leg

Leg	Dorsal cortex, mm	Lateral cortex, mm	Medial cortex, mm	Palmar cortex, mm
Left	5.15	5.44	5.62^{x}	4.12
Right	5.11	5.35	5.40^{y}	4.01
n	24	24	24	24
SEM	0.11	0.16	0.15	0.11
P – Value	0.48	0.40	0.06	0.21

^{x,y} Values lacking common superscripts within a column tend to differ ($P < 0.06$)
